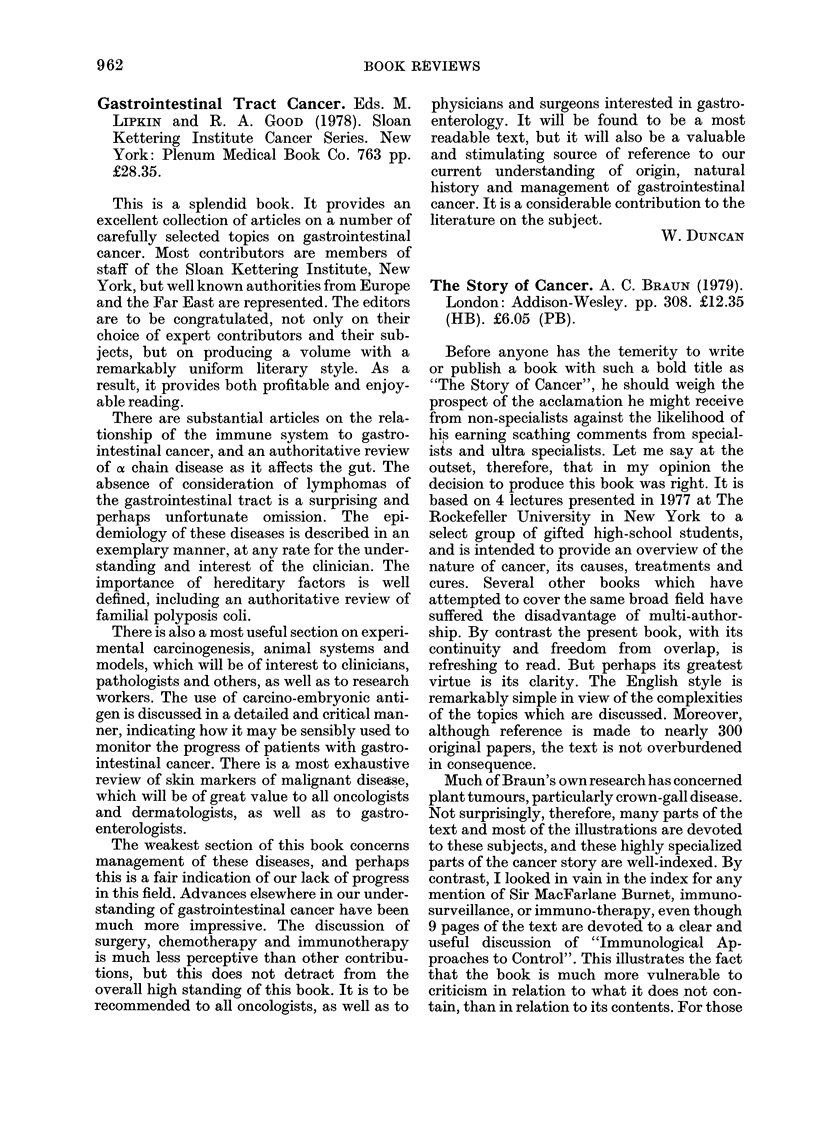# Gastrointestinal Tract Cancer

**Published:** 1979-12

**Authors:** W. Duncan


**Gastrointestinal Tract Cancer.** Eds. M. Lipkin and R. A. Good (1978). Sloan Kettering Institute Cancer Series. New York: Plenum Medical Book Co. 763 pp. £28.35.

This is a splendid book. It provides an excellent collection of articles on a number of carefully selected topics on gastrointestinal cancer. Most contributors are members of staff of the Sloan Kettering Institute, New York, but well known authorities from Europe and the Far East are represented. The editors are to be congratulated, not only on their choice of expert contributors and their subjects, but on producing a volume with a remarkably uniform literary style. As a result, it provides both profitable and enjoyable reading.

There are substantial articles on the relationship of the immune system to gastrointestinal cancer, and an authoritative review of $\alpha$ chain disease as it affects the gut. The absence of consideration of lymphomas of the gastrointestinal tract is a surprising and perhaps unfortunate omission. The epidemiology of these diseases is described in an exemplary manner, at any rate for the understanding and interest of the clinician. The importance of hereditary factors is well defined, including an authoritative review of familial polyposis coli.

There is also a most useful section on experimental carcinogenesis, animal systems and models, which will be of interest to clinicians, pathologists and others, as well as to research workers. The use of carcino-embryonic antigen is discussed in a detailed and critical manner, indicating how it may be sensibly used to monitor the progress of patients with gastrointestinal cancer. There is a most exhaustive review of skin markers of malignant disease, which will be of great value to all oncologists and dermatologists, as well as to gastroenterologists.

The weakest section of this book concerns management of these diseases, and perhaps this is a fair indication of our lack of progress in this field. Advances elsewhere in our understanding of gastrointestinal cancer have been much more impressive. The discussion of surgery, chemotherapy and immunotherapy is much less perceptive than other contributions, but this does not detract from the overall high standing of this book. It is to be recommended to all oncologists, as well as to physicians and surgeons interested in gastroenterology. It will be found to be a most readable text, but it will also be a valuable and stimulating source of reference to our current understanding of origin, natural history and management of gastrointestinal cancer. It is a considerable contribution to the literature on the subject.

W. Duncan